# Sentinel lymph node biopsy in gynecologic oncology: evidence-based recommendations for clinical practice - Number 13 - 2026

**DOI:** 10.61622/rbgo/2026FPS13

**Published:** 2026-10-13

**Authors:** Eduardo Batista Cândido, Matheus Eduardo Soares Pinhati, Ricardo dos Reis, Suzana Pessini, Ana Carla Ubinha, Agnaldo Lopes da Silva

**Affiliations:** Universidade Federal de Minas Gerais Belo Horizonte MG Brazil Universidade Federal de Minas Gerais, Belo Horizonte, MG, Brazil.; Faculdade de Ciências Médicas Belo Horizonte MG Brazil Faculdade de Ciências Médicas, Belo Horizonte, MG, Brazil.; Universidade Estadual Paulista Botucatu SP Brazil Universidade Estadual Paulista, Botucatu, SP, Brazil.; Hospital de Câncer de Barretos Barretos SP Brazil Hospital de Câncer de Barretos, Barretos, SP, Brazil.; Universidade Federal de Ciências da Saúde de Porto Alegre Porto Alegre RS Brazil Universidade Federal de Ciências da Saúde de Porto Alegre, Porto Alegre, RS, Brazil.; Hospital de Câncer de Barretos Barretos SP Brazil Hospital de Câncer de Barretos, Barretos, SP, Brazil.; Universidade Federal de Minas Gerais Belo Horizonte MG Brazil Universidade Federal de Minas Gerais, Belo Horizonte, MG, Brazil.

## Key points

- Sentinel lymph node (SLN) biopsy has become a cornerstone of modern surgical staging in gynecologic oncology.
- Robust evidence supports SLN biopsy as the preferred nodal staging in early-stage cervical, endometrial, and vulvar cancers when strict technical criteria are fulfilled.
- The use of indocyanine green (ICG) with near-infrared imaging provides the highest global and bilateral detection rates.
- Standardized surgical algorithms and mandatory ultrastaging are essential to ensure diagnostic accuracy and oncologic safety.
- Compared with systematic lymphadenectomy, SLN biopsy significantly reduces surgical morbidity without compromising oncologic outcomes.
- In ovarian cancer, SLN biopsy remains investigational and should not be adopted in routine clinical practice outside research protocols.

## Recommendations

- Sentinel lymph node biopsy is recommended as the preferred method for nodal staging in early-stage cervical cancer, provided that bilateral mapping is achieved and standardized algorithms are followed.
- In endometrial cancer, SLN biopsy using cervical injection of indocyanine green should be adopted as standard practice, including in patients with high-risk histologies, when performed by experienced teams and accompanied by ultrastaging.
- In early-stage vulvar cancer, SLN biopsy is recommended for selected patients with unifocal tumors smaller than 4 cm and clinically negative lymph nodes and should be performed exclusively in referral centers with expertise in the technique.
- Systematic lymphadenectomy should be avoided when SLN mapping is technically adequate, given the lack of survival benefit and the increased risk of postoperative morbidity.
- Strict adherence to validated surgical algorithms is mandatory, including side-specific pelvic lymphadenectomy in cases of mapping failure and resection of any suspicious lymph nodes regardless of tracer uptake.
- Ultrastaging with serial sectioning and immunohistochemistry is required in all SLN specimens to maximize detection of low-volume metastasis.
- Sentinel lymph node biopsy in ovarian cancer should not be recommended for routine practice and must be restricted to research settings until further evidence is available.

## Background

Sentinel lymph node (SLN) biopsy is based on the principle that lymphatic dissemination of solid tumors follows an orderly pathway, with the first draining lymph node reflecting the status of the entire nodal basin.^( 1 , 2 )^ After its validation in melanoma and breast cancer, SLN biopsy safely replaced radical nodal dissections, significantly reducing surgical morbidity without compromising oncologic outcomes.^( 3 , 4 )^

In gynecologic oncology, lymph node status is a key prognostic factor in cervical, endometrial, and vulvar cancers. Historically, systematic lymphadenectomy was adopted for surgical staging; however, most patients with early-stage disease do not harbor nodal metastases and therefore derive limited benefit from extensive nodal dissection.^( 5 )^ Moreover, lymphadenectomy is associated with relevant short- and long-term morbidity, including lymphedema, lymphocyst formation, neurovascular injury, and impaired quality of life.^( 6 )^ Randomized clinical trials in early-stage endometrial cancer failed to demonstrate a survival benefit from routine pelvic lymphadenectomy, despite increased detection of nodal disease, reinforcing concerns regarding overtreatment.^( 6 , 7 )^ These findings supported the development of less morbid staging strategies capable of maintaining diagnostic accuracy.

SLN biopsy was introduced in gynecologic oncology in the 1990s, initially in vulvar and cervical cancer, and progressively evolved through advances in tracer technology, imaging platforms, and surgical technique.^( 8 , 9 )^ The introduction of indocyanine green (ICG) with near-infrared fluorescence imaging, combined with standardized surgical algorithms and mandatory ultrastaging, significantly improved detection rates and reduced false-negative results. ^( 10 - 12 )^ As evidence accumulated, prospective studies and randomized trials demonstrated that SLN biopsy provides nodal staging accuracy comparable to systematic lymphadenectomy in selected gynecologic cancers, with substantially lower surgical morbidity. Consequently, SLN biopsy has been incorporated into international guidelines as the preferred approach for nodal assessment in early-stage cervical and endometrial cancers and as standard of care in selected cases of vulvar cancer.^( 13 - 15 )^ In contrast, the role of SLN biopsy in ovarian cancer remains investigational due to complex lymphatic drainage patterns, variable detection rates, and lack of robust survival data, limiting its use to research settings.^( 16 )^

Based on this evidence, FEBRASGO issues this Position Statement to provide clear, evidence-based guidance on the appropriate use of SLN biopsy in gynecologic oncology, supporting standardized practice and informed clinical decision-making.

## What is the current role of sentinel lymph node biopsy in gynecologic oncology?

Sentinel lymph node (SLN) biopsy is currently established as a validated strategy for nodal staging in gynecologic oncology, particularly in cervical, endometrial, and vulvar cancers. Its primary role is to provide accurate assessment of nodal status while reducing the morbidity associated with systematic lymphadenectomy, without compromising oncologic outcomes in appropriately selected patients.^( 2 - 4 )^

In early-stage cervical cancer, SLN biopsy has demonstrated high diagnostic accuracy when bilateral mapping is achieved and standardized algorithms are followed. Prospective studies report overall detection rates above 95%, with sensitivities ranging from 92% to 98% and negative predictive values exceeding 98% in patients with bilateral sentinel node detection.^( 13 , 17 , 18 )^ Randomized data further demonstrate a significant reduction in lymphatic morbidity when complete lymphadenectomy is omitted, with reported decreases in lymphatic complications from approximately 50% to 30%.^( 14 )^ In the PHENIX Trial, published in 2025, the 3-year disease-free survival was 96.9% in the SLN biopsy-only group and 94.6% in the lymphadenectomy group, and the cancer-specific survival at 3 years favored the SLN biopsy-only group (99.2% vs. 97.8%). Also, SLN alone was associated with substantially fewer surgical complications, including lower rates of lymphocyst, lymphedema, paresthesia, and pain.^( 19 )^ In endometrial cancer, SLN biopsy has progressively replaced routine pelvic lymphadenectomy as the preferred method for nodal staging. Large prospective trials using cervical injection of indocyanine green (ICG) reported sensitivities of 97-99% and negative predictive values above 99%, supporting the oncologic safety of SLN-only approaches.^( 15 )^ Importantly, SLN biopsy has shown comparable diagnostic performance in patients with high-risk histologies, while significantly reducing surgical morbidity.^( 20 )^

In vulvar cancer, SLN biopsy is the standard of care in carefully selected patients with early-stage disease, characterized by unifocal tumors confined to the vulva, smaller than 4 cm, with stromal invasion >1 mm and clinically negative groin nodes. Landmark trials demonstrated groin recurrence rates of approximately 2-3% following exclusive SLN biopsy, comparable to those observed after inguinofemoral lymphadenectomy, with substantially lower rates of long-term morbidity.^( 21 , 22 )^

Despite its broad adoption in these tumor sites, the role of SLN biopsy is not uniform across all gynecologic malignancies. In ovarian cancer, available studies report variable detection rates and sensitivities, and up to 25-30% of nodal metastases may not be identified by SLN mapping, precluding its routine clinical use outside research protocols.^( 16 )^

In gynecologic oncology, SLN accuracy must be interpreted per hemipelvis rather than per patient, reflecting the lateralized and asymmetric patterns of lymphatic drainage. This principle underpins the mandatory use of side-specific surgical algorithms and guides the interpretation of mapping success and failure. Overall, the current role of SLN biopsy in gynecologic oncology is that of a less morbid, evidence-based alternative to systematic lymphadenectomy for nodal staging in selected cancers, contingent upon appropriate patient selection, standardized surgical techniques, and quality-controlled pathological assessment.

## Which mapping techniques and tracers provide the best performance?

The performance of SLN biopsy in gynecologic oncology is highly dependent on the tracer used, the injection technique, and the imaging modality. Optimal mapping aims to achieve high overall detection, high bilateral detection, and a low false-negative rate, which are essential to ensure oncologic safety and allow omission of systematic lymphadenectomy.^( 11 )^

Early SLN mapping techniques relied on blue dyes and radioisotopes, either alone or in combination. Vital blue dyes, including patent blue and methylene blue, demonstrated overall detection rates ranging from 80% to 90%, but were consistently associated with lower bilateral detection rates and higher rates of mapping failure, particularly in obese patients.^( 23 , 24 )^ In addition, blue dyes are associated with rare but potentially severe allergic reactions and limited tissue penetration.^( 25 )^

Radioisotope-based mapping using technetium-99m–labeled nanocolloid improved detection rates and allowed preoperative lymphoscintigraphy. When combined with blue dye, overall detection rates of 89-92% and sensitivities close to 90% have been reported in cervical cancer. ^( 18 , 26 )^ However, the need for nuclear medicine infrastructure, higher costs, logistical complexity, and limited availability restrict its widespread use, particularly in low- and middle-income settings.^( 27 )^

The introduction of indocyanine green (ICG) with near-infrared fluorescence imaging represented a major advance in SLN mapping. Prospective studies and comparative trials consistently demonstrated superior performance of ICG compared with blue dyes and at least equivalent, and often superior performance compared with radioisotopes. Reported overall detection rates with ICG reach up to 96%, with bilateral detection rates ranging from 60% to 88% in cervical and endometrial cancers.^( 10 , 24 , 28 , 29 )^

Direct comparative studies further highlight the advantage of ICG. In one prospective comparison, bilateral detection was significantly higher with ICG than with methylene blue (88% vs. 55%), while overall detection approached 100% with fluorescence imaging.^( 24 )^ The randomized FILM trial confirmed the superiority of near-infrared fluorescence over patent blue dye, demonstrating higher detection rates and improved bilateral mapping with ICG.^( 29 )^ Importantly, ICG has shown better performance in patients with higher body mass index, a population traditionally associated with lower mapping success.^( 28 )^ It is advisable, whenever possible, to perform intraoperative frozen-section analysis of the SLN to prevent empty lymph node packets, particularly when SLN metastasis is suspected. In cases of mapping failure in one or both hemipelves, reinjection of the tracer is recommended before proceeding to systematic pelvic lymphadenectomy.

Injection site and technique are also critical determinants of mapping success. In cervical cancer, submucosal cervical injection at standardized positions, typically at 3 and 9 o'clock with superficial (with or without deep) infiltration, achieves the highest and most reproducible detection rates.^( 30 )^ In endometrial cancer, although hysteroscopic and fundal injections may increase para-aortic detection, cervical injection with ICG has emerged as the preferred technique due to its simplicity, reproducibility, and high pelvic detection rates, with sensitivity exceeding 95% when combined with standardized algorithms and ultrastaging.^( 15 , 31 )^

In vulvar cancer, peritumoral injection using radioisotope combined with blue dye remains the reference standard. Large prospective trials demonstrated detection rates above 95%, with negative predictive values exceeding 96% in properly selected patients.^( 21 , 22 )^ Emerging data suggest that ICG, alone or in combination with radioisotopes, may achieve comparable detection rates in experienced centers, although further validation is required.

Overall, current evidence supports ICG with near-infrared fluorescence imaging as the tracer of choice for SLN mapping in cervical and endometrial cancers, based on superior overall and bilateral detection rates, favorable safety profile, and operational feasibility. Regardless of the tracer used, optimal performance depends on standardized injection techniques and integration within validated surgical algorithms.

With regard specifically to ICG as the tracer for SLN, the concentration most commonly adopted in the literature is 1.25 mg/mL, as used in the FIRES trial.^( 15 )^ However, reported concentrations range from 0.5 mg/mL to 5 mg/mL, with variations in total injected volume and injection technique. The detection performance appears to be more strongly influenced by standardized technique and surgical expertise than by modest differences in ICG concentration.

## Why are standardized surgical algorithms essential?

Standardized surgical algorithms are essential to ensure the oncologic safety of SLN biopsy in gynecologic oncology. Although SLN mapping offers high diagnostic accuracy, its reliability depends on strict adherence to predefined technical steps designed to minimize false-negative results and compensate for mapping failures.^( 11 , 30 , 32 , 33 )^

Early studies demonstrated that SLN biopsy performed without a standardized algorithm was associated with unacceptable false-negative rates, particularly in cases of unilateral mapping or atypical lymphatic drainage. In cervical cancer, implementation of a side-specific algorithm, including mandatory removal of suspicious nodes and completion lymphadenectomy in cases of mapping failure, reduced false-negative rates from approximately 15% to 2%, while maintaining high sensitivity and negative predictive value.^( 11 )^

Prospective trials further confirmed the importance of algorithm-based approaches. In the SENTICOL study, no false-negative results were observed in patients with bilateral SLN detection, whereas false negatives occurred almost exclusively in cases with unilateral or failed mapping.^( 13 )^ Subsequent randomized data from SENTICOL II demonstrated that strict algorithm adherence allowed safe omission of complete lymphadenectomy, with a significant reduction in postoperative morbidity, including lymphatic complications decreasing from 51.5% to 31.4% and neurologic morbidity from 20.6% to 7.8%.^( 14 )^

In endometrial cancer, standardized SLN algorithms have shown similar impact. Retrospective application of a validated algorithm reduced false-negative rates from 15% to less than 2%, while prospective studies reported sensitivities above 95% and negative predictive values exceeding 99% when side-specific lymphadenectomy was performed in cases of mapping failure.^( 15 , 34 )^ Importantly, algorithm-based SLN biopsy also identified nodal metastases located outside traditional lymphadenectomy fields in up to 20–25% of cases, improving staging accuracy.^( 35 )^

Standardized algorithms also address anatomical variability and atypical drainage pathways. Studies have shown that SLN mapping reveals unexpected lymphatic drainage in approximately 10–15% of patients with cervical or endometrial cancer, reinforcing the need for systematic pelvic space dissection and structured evaluation of both hemipelves.^( 36 )^

Overall, standardized surgical algorithms transform SLN biopsy from a mapping technique into a reliable staging strategy. Their consistent application is fundamental to reducing false-negative rates, ensuring reproducible outcomes across institutions, and allowing safe de-escalation of surgical treatment without compromising oncologic outcomes. The key technical requirements and unacceptable practices for SLN biopsy in cervical and endometrial cancer are summarized in Flowchart 1 .

**Flowchart 1:** Unified SLN algorithm

**Cervical Cancer & Endometrial Cancer Patient Selection** Indications Early-stage cervical cancer Apparent uterine-confined endometrial cancer Contraindications Bulky nodal disease on imaging Prior retroperitoneal lymphadenectomy Known hypersensitivity to ICG
**STEP 1 — Tracer Preparation** Use Indocyanine Green (ICG) as first-line tracer Near-infrared fluorescence imaging required ICG concentration: 1.25 mg/mL is most commonly used. Concentrations from 0.5–5 mg/mL have been reported
**STEP 2 — Cervical Injection (Standardized Entry Point)** Technique Inject at 3 and 9 o'clock Superficial stromal injection mandatory Optional deep component Use 20–25G needle Inject slowly with tissue resistance feedback **Important:** Cervical cancer: Avoid intratumoral injection Endometrial cancer: Do NOT perform transperitoneal uterine injection Do NOT perform hysteroscopic injection
**STEP 3 — Before Mapping** Perform white-light pelvic inspection If uterine manipulator is used → insert after injection
**STEP 4 — Retroperitoneal Access (Mandatory Landmarks)** Open paravesical and pararectal spaces and identify: Ureter External iliac vessels Internal iliac artery Obliterated umbilical artery Paravesical space No nodal dissection before full anatomic orientation.
**STEP 5 — Mapping and Channel Identification** Switch to near-infrared mode Identify fluorescent lymphatic channels Follow channels starting at uterine artery level Dissect laterally away from the uterus Complete one hemi-pelvis before moving contralaterally
**STEP 6 — SLN Identification** A node qualifies as SLN if: It is the first node receiving direct fluorescent drainage It is the most proximal node along the channel It may include immediate echelon node if contiguous
**STEP 7 — SLN Dissection Principles** Use blunt and/or energy-based technique Avoid disruption of lymphatic channels Isolate node before removal Grasp at lymphatic pedicle (not node center) Ensure full hemostasis
**STEP 8 — Labeling and Documentation** Label each SLN by: Side (Right/Left) Nodal location: Obturator External iliac Internal iliac Common iliac Presacral Paraaortic (if applicable)
**STEP 9 — Pathology Protocol** Initial H&E If negative → ultrastaging with serial sectioning ± IHC
**STEP 10 — Intraoperative Troubleshooting** If No Mapping on One Side: Option A Side-specific lymphadenectomy Option B Re-inject ICG Extend dissection to common iliac / presacral Map contralateral side first, then reassess If "Empty Packet" on Final Pathology: Consider reoperation only if nodal status alters adjuvant management

## How about de frozen section assessment?

In gynecologic malignancies, intraoperative frozen-section analysis of the SLN may assist surgical decision-making. A positive result can immediately modify the planned procedure, including completion lymphadenectomy or alteration of the primary surgical strategy. Frozen section also helps distinguish true SLNs from empty nodal packets, thereby improving procedural accuracy. Its use is particularly justified when there is intraoperative suspicion of nodal metastasis.^( 30 , 37 )^

However, frozen section has limited sensitivity for low-volume disease, especially micrometastases and isolated tumor cells, and should not be relied upon to exclude nodal involvement. Definitive histopathologic ultrastaging remains mandatory for accurate assessment in all patients undergoing SLN mapping.^( 30 )^

## What is the role of ultrastaging?

Ultrastaging plays a critical role in SLN biopsy by significantly increasing the detection of low-volume metastatic disease that would otherwise remain undetected by routine hematoxylin and eosin evaluation. Its incorporation into SLN protocols is essential to ensure diagnostic accuracy and oncologic safety, particularly when systematic lymphadenectomy is omitted.^( 12 , 38 )^

Multiple studies have demonstrated that ultrastaging increases the detection of nodal metastases by 10–20% compared with standard pathological assessment. In cervical cancer, ultrastaging has been shown to identify additional micrometastases and isolated tumor cells in up to 15–25% of patients initially classified as node-negative by conventional pathology.^( 39 , 40 )^ Similar findings have been reported in endometrial cancer, where ultrastaging increased nodal positivity rates by approximately 8–15%, particularly in high-risk histologic subtypes.

**Figure 1:**
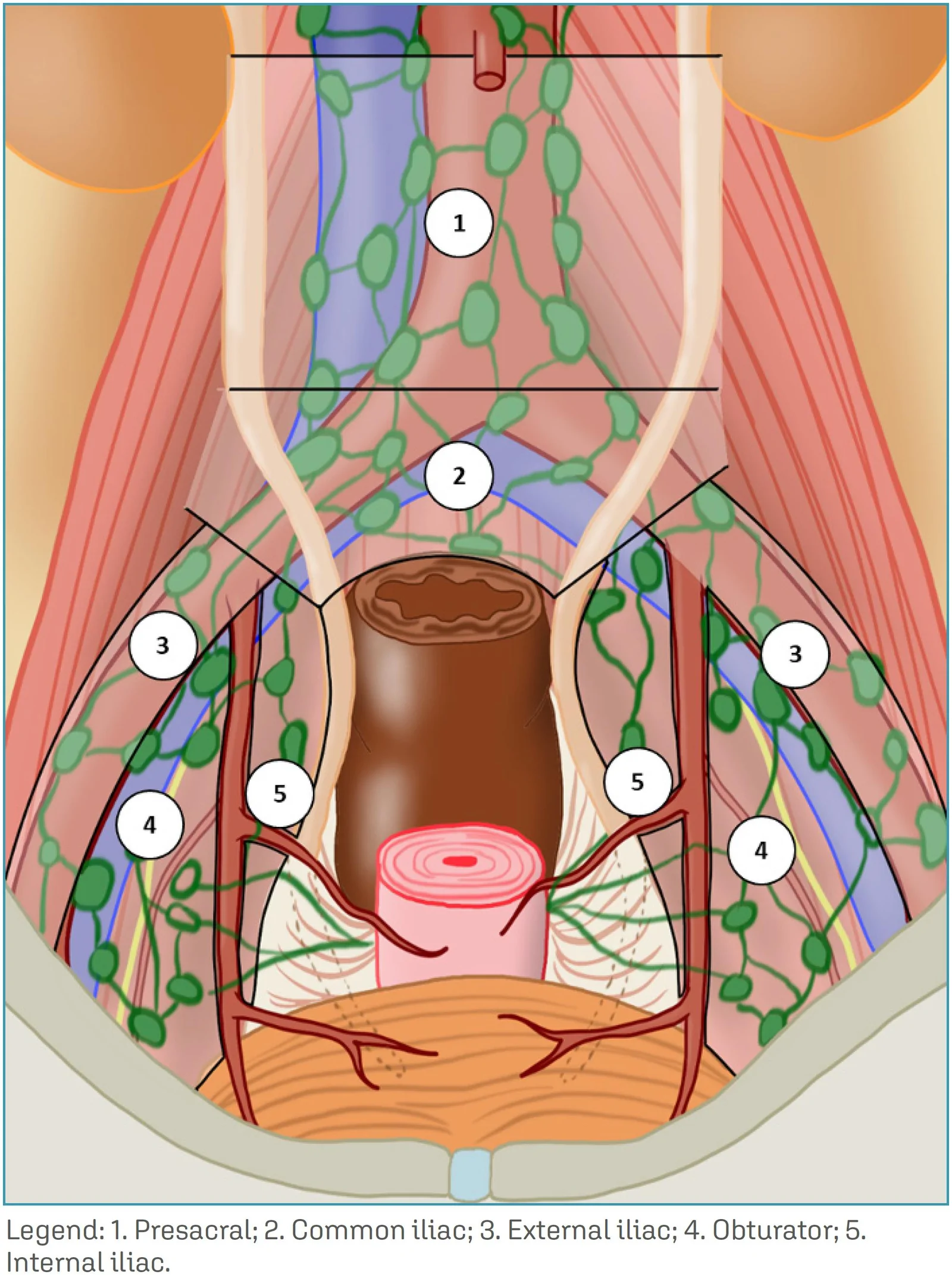
Lymphatic drainage of cervical and endometrial cancer

Prospective trials highlight the magnitude of this impact. In the SENTICOL study, ultrastaging accounted for the detection of nearly 40% of all nodal metastases identified, underscoring its importance in accurate staging.^( 13 )^ In the FIRES trial, more than 50% of metastatic SLNs contained low-volume disease detected exclusively through ultrastaging, contributing to the reported sensitivity of 97% and negative predictive value exceeding 99%.^( 15 )^

Beyond diagnostic yield, ultrastaging has relevant clinical implications. Although the prognostic significance of isolated tumor cells remains debated, several studies associate micrometastases detected by ultrastaging with an increased risk of recurrence, supporting their consideration in adjuvant treatment planning.^( 41 , 42 )^ Importantly, omission of ultrastaging has been associated with higher false-negative rates and potential understaging, particularly in patients with unilateral mapping or limited nodal involvement.^( 36 )^

Standardized ultrastaging protocols typically involve serial sectioning of SLNs at multiple levels combined with immunohistochemical staining for cytokeratins. This approach allows reliable detection of micrometastases while maintaining reproducibility across pathology laboratories. Current evidence supports mandatory ultrastaging of all SLNs as an integral component of SLN biopsy algorithms in gynecologic oncology.^( 32 )^

Overall, ultrastaging is not merely an adjunct but a fundamental element of SLN mapping. Its systematic application enhances diagnostic accuracy, reduces false-negative results, and supports safe de-escalation of surgical treatment, reinforcing its essential role in contemporary gynecologic oncology practice.

## How should SLN biopsy be applied according to tumor site?

The clinical application of SLN biopsy in gynecologic oncology must be guided by tumor-specific patterns of lymphatic spread, disease prevalence, and the quality of available evidence. Although the general principles of SLN mapping, tracer selection, standardized algorithms, and ultrastaging are shared across malignancies, their translation into clinical practice differs according to tumor site. This section summarizes the current evidence supporting site-specific use of SLN biopsy, highlighting indications, technical requirements, diagnostic performance, and limitations, with the objective of supporting consistent, evidence-based clinical decision-making.^( 11 , 32 , 43 , 44 )^ The current evidence and clinical status of SLN biopsy according to tumor site in gynecologic oncology is shown in Table 1 .

**Table 1:** Current evidence and clinical status of sentinel lymph node biopsy according to tumor site in gynecologic oncology

Tumor site	Clinical indication	Recommended tracer	Overall detection rate	Bilateral detection rate	False-negative rate *	Level of evidence
Cervical cancer	Early-stage disease (IA1–IB2)	ICG (preferred) ± radiotracer	∼95–98%	∼60–88%	∼2–5%	High (prospective trials, RCTs)
Endometrial cancer	Low- and high-risk histologies	ICG (cervical injection)	∼90–97%	∼50–80%	<3%	High (prospective trials)
Vulvar cancer	Unifocal tumors <4 cm, cN0	Radiotracer + blue dye (standard); ICG alternative	>95%	Not applicable	∼2–4%	High (prospective trials)
Ovarian cancer	—	Not established	Variable	Variable	Up to 25–30%	Low (feasibility studies)

* False-negative rates reported when standardized algorithms and ultrastaging are applied.

In early-stage cervical cancer, SLN biopsy is recommended as a standard approach for nodal staging when bilateral mapping is achieved and validated surgical algorithms are followed. High-level evidence from prospective and randomized studies demonstrates excellent diagnostic accuracy and significant reduction in surgical morbidity, supporting its routine clinical use in appropriately selected patients.^( 13 , 14 , 17 )^

In endometrial cancer, SLN biopsy using cervical injection of ICG has become the preferred method for nodal assessment across risk groups. Prospective trials have confirmed high sensitivity and negative predictive value, including in high-risk histologies, while consistently reducing lymphatic morbidity compared with systematic lymphadenectomy.^( 15 , 35 )^

In vulvar cancer, SLN biopsy is standard of care for selected patients with early-stage (< 4 cm), unifocal disease and clinically negative groins. Large prospective trials demonstrated low groin recurrence rates and substantial reductions in long-term morbidity, provided that strict selection criteria and pathological ultrastaging are applied.^( 21 , 22 )^

In contrast, SLN biopsy in ovarian cancer remains investigational. Complex and variable lymphatic drainage, inconsistent detection rates, and high false-negative rates currently preclude its adoption in routine clinical practice, restricting its use to research protocols.^( 16 )^

## Final Considerations

Sentinel lymph node (SLN) biopsy represents a paradigm shift in the surgical staging of gynecologic malignancies, allowing accurate nodal assessment while significantly reducing the morbidity historically associated with systematic lymphadenectomy. Accumulated evidence from prospective trials, randomized studies, and large multicenter cohorts supports its safe and effective use in cervical, endometrial, and vulvar cancers when strict technical and pathological standards are applied.

The oncologic safety of SLN biopsy is contingent upon a comprehensive approach that integrates appropriate patient selection, use of validated tracers, preferably ICG with near-infrared fluorescence imaging, standardized surgical algorithms, and mandatory pathological ultrastaging. When these elements are consistently applied, SLN biopsy achieves high detection rates, low false-negative rates, and excellent negative predictive values, enabling safe de-escalation of surgical treatment without compromising oncologic outcomes.

**Table 2:** Technical Do's and Don'ts for SLN biopsy in cervical and endometrial cancer

DO's (Recommended Technical Practices)	DON’Ts (Unacceptable Technical Practices)
Use ICG with near-infrared fluorescence imaging.	Do not perform SLN biopsy without a validated surgical algorithm.
Perform standardized cervical injection, preferably at 3 and 9 o'clock, with superficial (with or without deep) infiltration.	Do not use non-standard or non-validated injection techniques
Conduct systematic anatomical dissection of pelvic spaces prior to SLN identification.	Do not assess mapping success globally per patient, instead of per hemipelvis.
Evaluate SLN mapping by hemipelvis, not per patient.	Do not accept unilateral mapping as definitive nodal staging. If necessary, reinject the dye.
Excise the SLN as an isolated specimen, preserving surrounding lymphatic structures.	Do not omit side-specific lymphadenectomy when SLN mapping fails.
Remove any lymph node suspicious on inspection or palpation, regardless of tracer uptake.	Do not rely solely on intraoperative frozen section to exclude micrometastases.
Apply mandatory ultrastaging, including serial sectioning and immunohistochemistry, to all SLN specimens.	Do not perform SLN pathologic evaluation using hematoxylin–eosin staining alone.
Follow validated, side-specific algorithms, including completion lymphadenectomy when indicated.	Do not perform SLN biopsy without adequate surgical expertise and specialized pathological support.
Ensure the procedure is performed by experienced surgical teams with appropriate infrastructure.	Do not use SLN biopsy as an automatic substitute for lymphadenectomy outside validated indications.

Note: These technical recommendations apply to gynecologic malignancies in which SLN biopsy is a standardized and validated staging strategy with shared anatomical and algorithmic principles, namely cervical and endometrial cancers; SLN biopsy in vulvar cancer follows tumor-specific protocols and is addressed separately; SLN biopsy in ovarian cancer remains investigational and is not covered by these technical recommendations.

Importantly, SLN biopsy should not be viewed as a simplified or partial procedure. On the contrary, it requires surgical expertise, institutional experience, and multidisciplinary coordination, particularly with pathology services. Inadequate technique, incomplete adherence to algorithms, or omission of ultrastaging may undermine diagnostic accuracy and lead to understaging, reinforcing the need for quality control and structured training.

While SLN biopsy is firmly established in cervical, endometrial, and vulvar cancers, its role in ovarian cancer remains investigational. Current evidence does not support its routine clinical use in this setting, and its application should be restricted to research protocols until further data become available.

Based on the current body of evidence, FEBRASGO endorses SLN biopsy as a standard component of contemporary gynecologic oncology practice for selected tumor sites. This Position Statement aims to support evidence-based clinical decision-making, promote standardized practice across institutions, and encourage the responsible implementation of SLN biopsy as part of high-quality, patient-centered care.

## Data Availability

research data are available in the article.

## National Commission Specialized in Gynecologic Oncology of the Brazilian Federation of Gynecology and Obstetrics Associations (Febrasgo)

President:

Eduardo Batista Candido

Vice-president:

Renato Moretti Marques

Secretary:

Sophie Françoise Mauricette Derchain

Members:

Ana Carla Franco Ubinha
Caetano Da Silva Cardial
Cristina Anton
Delzio Salgado Bicalho
Etelvino De Souza Trindade
Geórgia Fontes Cintra
Jesus Paula Carvalho
Jose Carlos De Jesus Conceição
Leandro Santos De Araújo Resende
Marcia Luiza Appel Binda
Mariana Seabra Leite Praça
Ricardo Dos Reis
Suzana Arenhart Pessini
Walquiria Quida Salles Pereira Primo
